# A Severe Case of Cutaneous Adverse Drug Reaction Secondary to a Novice Drug: Idelalisib

**DOI:** 10.1177/2324709617711463

**Published:** 2017-05-24

**Authors:** Joseph Gabriel Gabriel, Aaysha Kapila, Alexei Gonzalez-Estrada

**Affiliations:** 1 East Tennessee State University, Johnson City, TN, USA

**Keywords:** idelalisib, adverse drug reaction, exfoliative dermatitis, chronic lymphocytic leukemia, phosphatidylinositol 3-kinase δ

## Abstract

Phosphatidylinositol 3-kinase δ (PIK3δ) is a tyrosine kinase essential for B cell survival, making it an important target in the treatment of chronic lymphocytic leukemia. Idelalisib is an inhibitor of PIK3δ demonstrating initial success in disease response, but is now shown to have a decreased overall survival and life-threatening serious adverse events. The following is an unfortunate case of a grade III adverse skin reaction secondary to idelalisib with the likely complication of methicillin-resistant *Staphylococcus aureus* bacteremia.

## Introduction

Important for B cell proliferation and homeostasis, phosphatidylinositol 3-kinase δ (PIK3δ) is a tyrosine kinase of interest in the treatment of chronic lymphocytic leukemia (CLL). As a specific PIK3δ inhibitor, the Gilead Sciences creation idelalisib (Zydelig), was approved by the US Food and Drug Administration (FDA) with breakthrough therapy designation on July 2014 for the treatment of relapsed CLL in combination with rituximab.^1,2^ However, ongoing Phase III clinical studies, later in the course, showed decreased overall survival and higher rates of serious adverse events (SAE). We provide a case of a male presenting with diffuse exfoliative rash secondary to idelalisib and complications of methicillin-resistant *Staphylococcus aureus* (MRSA) bacteremia.

## Case Report

A 65-year-old African American male was admitted to the hospital for a 6-day history of worsening generalized rash involving his mucous membranes. His medical history was significant for hepatitis B infection and CLL Rai Stage I. Prior treatment regimens included fludarabine, cyclophosphamide, and rituximab combination and ibrutinib alone. However, these were stopped due to varying adverse events. Consequently, the patient was started on idelalisib monotherapy 150 mg twice daily for 3 months. Off-label use of monotherapy was initiated as opposed to the FDA-approved combination therapy due to concerns of reactivation of hepatitis B with rituximab, which was discovered later in the course of his disease. Nevertheless, with the development of a desquamating rash starting at the soles of his feet, idelalisib was discontinued 2 weeks prior the current admission.

**Figure 1.**
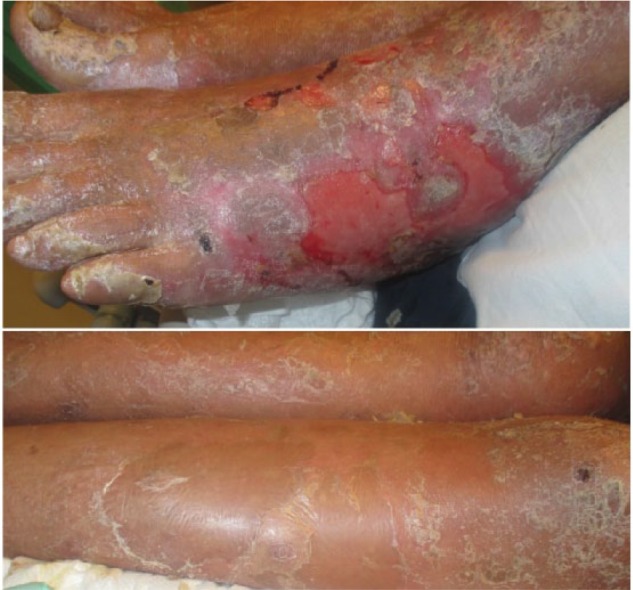
Severe cutaneous reaction of the lower extremities present approximately 2 weeks after the last dose of idelalisib. *Top panel*: Exfoliation of the dermis of the foot. *Bottom panel*: Concurrent scaling of the lower legs.

Examination revealed an exfoliative and erythematous rash throughout his torso, extremities (Figure 1), tongue, and glans penis. He had diffuse erythema and scaling with lesions that were found to be in various stages of healing. Of note, the Nikolsky sign was negative. Hepatic function on routine monitoring 16 days prior initiation of idelalisib were aspartate aminotransferase (AST) 18 U/L (normal = 15-46 U/L) and alanine aminotransferase (ALT) 16 U/L (normal = 7-56 U/L). However, at the time of admission, laboratory work showed elevated liver enzymes (AST 60 U/L, ALT 106 U/L), leukocytosis (17.8 × 10^3^/µL [normal = 4.8-10.5 × 10^3^/µL), and a mild normocytic anemia (Hgb 10.9 g/dL [normal = 13.6-17.3 g/dL], mean corpuscular volume 82.5 fL [normal = 83.5-96.8 fL]). At first, he was thought to have toxic epidermal necrolysis, supported by the temporal association with idelalisib. During his hospitalization, however, with its protracted course and clinical diagnosis of diffuse erythema and scaling involving ≥90% body surface area, the patient was classified as having idelalisib-induced exfoliative dermatitis secondary to a severe cutaneous adverse drug reaction.

Unfortunately, a biopsy, which would have further classified this reaction, could not be completed due to the rapid decline of his hospital course. This included respiratory failure requiring intubation and MRSA-induced septic shock. A contrast computed tomography scan revealed axillary and mediastinal lymphadenopathy, with a large consolidation in the left upper lobe. He underwent a thoracotomy for decortication and assessment of the mass showed high-grade lymphoma. Ultimately, his family decided to pursue hospice care and the patient was terminally extubated.

## Discussion

Primarily expressed in leukocytes as a tyrosine kinase, PIK3δ is essential in BCR signaling for the promotion of B cell survival and proliferation.^3^ Idelalisib, a specific inhibitor of PIK3δ, was approved by the US FDA for the treatment of relapsed CLL.^1,2^ Initial registration trials, when combined with rituximab, an anti-CD20 monoclonal antibody, showed promising results including increased progression-free survival (PFS) with equal rates of adverse events in comparison to rituximab alone. Its overall response rate (ORR) in Phase III studies was 83% with a PFS of 24 months compared to an ORR of only 13% and a PFS of 7.3 months seen with rituximab and placebo.^1^ However, in May 2016, 7 clinical trials were discontinued due to decreased overall survival and higher rates of SAE with idelalisib. The most common SAE, which are now found as a black-box warning, include life-threatening hepatotoxicity, colitis, intestinal perforation, and pneumonitis.^2^ Interestingly, only 2% of patients in recent trials developed Grade ≥3 rash and only one case of toxic epidermal necrolysis. To our knowledge, this is one of the first few cases of idelalisib-induced severe cutaneous adverse drug reaction that can be found in the current literature. Treatment is mainly supportive with discontinuation of the drug.^1^ For more severe cases of other complications, like pneumonitis and colitis, systemic corticosteroids can be used.

## Conclusion

This illustrates an uncommon, but severe case of adverse cutaneous reaction to idelalisib. Current evidence on the mechanism of this complication, and its more common SAE, remain limited and continued research to elucidate this pathophysiology is important not only for medical knowledge but also to help establish management strategies.^2^ Nevertheless, PIK3δ continues to be an important target for research in the treatment of CLL.
